# Nutritional Status and Associated Risk Factors of Syrian Children’s Residents in the Kingdom of Saudi Arabia

**DOI:** 10.3390/children8111053

**Published:** 2021-11-15

**Authors:** Khaled M. AlZoubi, Ghedeir M. Alshammari, Abdulrahman S. AL-Khalifah, Mohammed A. Mohammed, Hind E. Aljuhani, Mohammed A. Yahya

**Affiliations:** Department of Food Science and Nutrition, College of Food and Agricultural Science, King Saud University, P.O. Box 2460, Riyadh 11451, Saudi Arabia; khaledalzoubi7@gmail.com (K.M.A.); akhalifa@ksu.edu.sa (A.S.A.-K.); Muhammed-awad@hotmail.com (M.A.M.); hind.e.j@hotmail.com (H.E.A.); mabdo@ksu.edu.sa (M.A.Y.)

**Keywords:** Syrian respondents, malnutrition, overweight, stunting, Saudi Arabia, nutritional awareness

## Abstract

This study aimed to evaluate the nutritional status and associated risk factors of Syrian children living in Saudi Arabia. In this study, 55 boys and 55 girls ranging in age from 6 to 12 years were selected. Socioeconomic data of families were collected using a structured questionnaire. The participants’ anthropometric measurements were calculated. The 24-h recall method was applied to calculate the daily food intake. Dietary nutrients’ average daily intake of both boys and girls was significantly (*p* ≤ 0.01) lower than that of the dietary reference intake (DRI) with few exceptions. The sedentary lifestyles of both boys and girls had a negative impact on their food choices, and as a result, a large number of them were underweight and suffered from malnutrition and stunting. Spearman correlation coefficients revealed that most of the children’s dependent variables were risk factors and strongly and negatively associated with their nutritional status proxies. The study concluded that both boys and girls had unbalanced nutritional status with high percentages of malnutrition and stunting because most dependent factors were adversely related to the independent ones.

## 1. Introduction

Syrian families living in Saudi Arabia are lawfully residing as displaced families, allowing them to work and live or legally residing. Displaced families are expected to encounter numerous obstacles affecting their health, livelihoods, and survival, including food and nutrition inadequacy. However, children are a particularly vulnerable population group, susceptible to conflict, poverty, and food insecurity. Dietary inadequacy may negatively impact their height, physical ability, and psychosomatic development at this stage in their lives. It may increase the risk of certain diseases (including cardiovascular disease) and even death in adult life [1,2]. These issues can impact children’s educational success and their future economic output [3]. In addition, school-aged children are more likely to miss breakfast, eat less fruit and vegetables, and engage in unhealthy snacking habits that might negatively impact their nutritional status and raise their risk of obesity and co-morbidities [4].

The assessment of nutritional status is a tool by which a nutritionist can evaluate a patient to help maintain average growth and health, identify risk factors that contribute to disease, and detect malnutrition and nutritional deficiencies early so that they can be treated. Following an individual during childhood with standardized criteria provides the basis for sound recommendations and evaluation of nutritional therapy [5,6]. Physical growth is a complex process influenced by environmental, genetic, and dietary factors [7]. Comparison with standard age and gender references helps identify abnormalities in growth and development that may be due to nutrient deficiencies or excesses [8]. The three most commonly used anthropometric indices in children are weight-for-height, height-for-age, and weight-for-age [9]. During primary school education (6–12 years), the average weight and height increase annually by 3.2 kg and 6.3 cm, respectively [10].

Factors that affect children’s growth and nutritional status include changes in nutrient intake, disease, socioeconomic status, and regional developments [11,12,13,14]. Inappropriate nutrition could be the leading cause of obesity and many other diseases [15]. There is an increase in the prevalence of certain diseases associated with food and bad dietary habits in Saudi Arabia, such as obesity, high blood pressure, and diabetes [16]. With low consumption of dietary fiber, vegetables, and fruits and a high intake of fat, sugar, and salt, the Western diet has bypassed the traditional eating patterns of Saudi Arabia. Thus, the nutritional status of Syrian children, whether they are displaced or live in a legal residence, is expected to be changed drastically. Therefore, the present study was conducted to assess the nutritional status and associated risk factors of Syrian children aged 6 to 12 years who reside in Saudi Arabia.

## 2. Materials and Methods

### 2.1. Study Sample

A convenient sample of 110 Syrian boys and girls (55 each) living in Riyadh, Saudi Arabia, as displaced or legally (both with an opportunity to work and live) aged 6 to 12 years, was selected. Most of the respondents’ families have been in Saudi Arabia for more than five years. A cross-sectional survey was conducted between May and October 2019. Participants were recruited through social visits, schools, nurseries, and open spaces. The father or mother can answer the questions for boys and girls who cannot communicate with. Respondents are recruited according to age, nationality, and lack of chronic diseases. Accordingly, the number of respondents does not exceed 55 boys and 55 girls.

### 2.2. Socioeconomic and Anthropometric Data

Household socioeconomic data and dietary habits were collected using a structured questionnaire described by Khayri et al. [17]. Anthropometric data were used to assess the nutritional status of children according to WHO classification [18]. Height was measured to the nearest 0.5 cm using a Detecto stadium scale (Detecto, Webb City, MO, USA) without shoes and body weight to the nearest 0.1 kg on a balance beam scale (Seca Corp Scale, Deutschland, Germany) with subjects wearing light clothing and without shoes.

The indicators of respondents’ nutritional status were obtained using the following equations:

$$\mathrm{Height}-\mathrm{for}-\mathrm{age}=\frac{\mathrm{Height}\;\mathrm{of}\;\mathrm{the}\;\mathrm{pupil}}{\mathrm{Height}\;\mathrm{of}\;\mathrm{reference}\;\mathrm{pupil}\;\mathrm{in}\;\mathrm{the}\;\mathrm{same}\;\mathrm{age}}\;\times \;100$$

$$\mathrm{Weight}-\mathrm{for}-\mathrm{age}=\frac{\mathrm{Weight}\;\mathrm{of}\;\mathrm{the}\;\mathrm{pupil}}{\mathrm{Weight}\;\mathrm{of}\;\mathrm{reference}\;\mathrm{pupil}\;\mathrm{in}\;\mathrm{the}\;\mathrm{same}\;\mathrm{age}}\;\times \;100$$

$$\mathrm{BMI}=\frac{\mathrm{Weight}\;\mathrm{in}\;\mathrm{kg}}{{\left(\mathrm{Height}\;\mathrm{in}\;M\right)}^{2}}$$

The height and weight standards were obtained as described in WHO [18].

### 2.3. Determination of Nutritional Habits and Nutrient Intake

Dietary habits data included the number of meals/day, breakfast, preferred food, special diet, vitamins and supplements, and intake of soft drinks and sweets. In the case of nutrient intake, participants were asked to give an idea of what meal they had eaten each day. The 24 h food recall data for each respondent were entered into the ESHA food processor program. Next, the nutrients taken were compared with the dietary reference intakes (DRI) [19]. 

### 2.4. Statistical Analysis 

Statistical analysis was performed with the SPSS V. 21 software. All data collected from respondents were coded for subsequent computer processing and analysis. Frequency and descriptive statistics were obtained for all variables. In addition, a student *t*-test was used to compare children’s food intake (24 h recall) and DRI. Spearman correlation coefficients between the children’s nutritional status as a dependent variable and some hypothesized independent variables were calculated to determine the nutritional risk factors.

### 2.5. Ethical Considerations

All study procedures were approved by Institutional Review Board, King Saud University, Riyadh, Saudi Arabia (IRB. Ref. No: KSU- HE-19- 130, date of approval 5 March 2019). Additionally, the authors would like to indicate the following:

1. The study does not involve any threat or invasion of the respondents’ family privacy.
2. Before data collection, the respondents and their families were fully informed by the nature and objective of the study.
3. After explaining the study objectives and procedure, the children’s parents obtained verbal and written informed consent. Questionnaires were filled out with the help of their parents.

## 3. Results

### 3.1. Frequency Distribution of Participants According to Socioeconomic Characteristics and Nutritional Habits 

As shown in Table 1, 58.18% of boys and 74.55% of girls belong to families with more than five members. The educational level of the boys’ father was secondary (32.73%) or university (40%), while that of the girls was also secondary (43.64%) or university (30.31%). Most participants’ families had an average monthly income (40% of boys and 54.54% of the girls’ families), and a small number (7.27% of boys and 12.73% of the girls’ families) had a high monthly income. More than 70% and 80% of the boys’ and girls’ mothers, respectively, did not engage in work. The frequency distribution of Syrian participants according to their food habits is shown in Table 2. Most of the boys and girls (65.5% and 63.64%) ate three meals daily, and most ate breakfast regularly (96.36% and 98.18%), with only a few exceptions. About 60% of the boys and 52.73% of the girls liked specific food, but the rest preferred to take all kinds of food provided. All participants did not follow a special diet regime. More than 85% of the participants did not take vitamins or supplements, but 50% drank soft drinks, and 20% ate sweets.

### 3.2. Average Daily Nutrient Intake Concerning DRI

The nutritional status of children was assessed by investigating their intake of nutrients and compared with the DRI (Table 3). Most of the participants’ average daily nutrient intakes were significantly lower (*p* ≤ 0.01) than the DRI, except for protein, unsaturated fats, several vitamins (B2 and B12), and selenium, which were significantly higher (*p* ≤ 0.01 or *p* ≤ 0.05) than the DRI. The mean daily cholesterol intake was significantly lower than the DRI (*p* ≤ 0.01) and daily intakes of vitamin D, calcium, and iron.

### 3.3. Anthropometric Characteristics of Participants

Table 4 shows the BMI of the participants. The results showed that 21.82% of boys and 41.82% of girls were underweight, while 69.09% of boys and 49.09% of girls were of normal nutritional status. About 5.45% of boys are overweight, compared to 7.27% of girls, with both having a lower obesity rate. The weight-for-age and height-for-age of the participants were also determined, and the results are presented in Table 5. According to the data, 49.09% of boys and 70.91% of girls suffered from very low wasting, while the rest had low, medium, high, or very high wasting. Severe wasting was high among boys (9%) compared to girls (7%). About 80% of boys suffered from very low stunting, compared to 58.18% of girls. The rate of stunting among the rest was low, medium, high, or very high. However, severe stunting was observed among girls (9.09%) compared to boys (1.82%). 

### 3.4. Risk Factors Associated with Participants’ Nutritional Status

Spearman’s correlation coefficients were calculated between participant family’s socioeconomic characteristics, dietary habits, and anthropometrics (Table 6). The anthropometric characteristics (BMI, height-for-age, and weight-for-age) were directly or inversely correlated with the participants’ socioeconomic factors and food habits. Participants’ BMI was directly and significantly correlated to their age, number of family members, father’s education, monthly income, number of meals per day, and soft drinks and sweets consumption. However, for boys, other variables such as mother’s education, mother’s work, breakfast intake, particular food preference, and special diet were inversely and significantly associated with BMI. In contrast, breakfast intake and special diet variables were inversely and significantly correlated with BMI for girls. For both sexes, age, family members, monthly income, intake of breakfast, soft drinks, and sweets, in addition to father’s education for girls, were inversely and significantly correlated with the participants’ weight-for-age (malnutrition). However, a particular diet regimen was directly and significantly associated with the participants’ weight-for-age. Most of the independent variables of the participants were either positively or negatively correlated (*p* ≤ 0.01, *p* ≤ 0.05) with height-for-age data (stunting).

## 4. Discussion

A cross-sectional study was conducted to assess the nutritional status and associated risk factors among Syrian children residing in Saudi Arabia. Socioeconomic data of the participants’ families indicated that large families and traditional education for parents were characteristic for both sexes. This reflects the Syrian culture that favors large families. Maternal education appears to be an essential factor in the infant’s nutritional situation, and the relationship between increased maternal education and child survival and development is consistent and robust [20]. The mother’s education is essential and allows her to use health services better and provide better childcare, especially breastfeeding. Khayri et al. [21] studied the association between some socioeconomic characteristics, food habits, and food intake of Sudanese primary school pupils in Riyadh City, Kingdom of Saudi Arabia, and reported similar findings.

The father’s education is also essential for proper child nutrition because, in Saudi Arabia, the family’s primary income earner is primarily male [21]. Therefore, the majority of the participants’ mothers did not have a job. A high percentage of participants had three meals breakfast daily, and a very low rate skipped breakfast. It has been reported that the relatively low intake of vitamins, minerals, and other nutrients by breakfast-skippers could not be replaced by any meal of the day [21,22]. The reason for skipping breakfast could be due to a lack of appetite in the early morning, or it may be a habit practiced by the family [17]. The current results showed that a high percentage of the participants prefer special food, which agrees with Al-Aqeel [23], who reported that an equal percentage of participants preferred to take certain foods. Their family or caregiver strongly influences children’s eating habits and preference for specific foods during childhood [24]. None of the participants had a special diet regime due to a lack of knowledge about the diet regimen benefits. This finding agrees with a study conducted with Sudanese primary school pupils in Riyadh City, Saudi Arabia [21]. The majority of participants consumed soft drinks and sweets either sometimes or always. The consumption of large amounts of sugar-added drinks and sweets by children is associated with poor eating habits, reduced intake of nutrients, and increased risk of obesity [21,25]. 

The majority of the respondents’ intake of energy foods was significantly lower than the DRI. The lower intake of fat and carbohydrates by participants living in Saudi Arabia decreased energy intake [17,21,26]. The lower percentage of saturated fats than unsaturated ones could be due to reduced animal protein intake and vegetable oils, which lowers animal fats, as reported by Ali et al. [26] for primary school pupils in Saudi Arabia. The decrease in iron intake could be due to a low intake of proteins [17]. Moreover, low consumption of fruits and vegetables decreased the intake of some vitamins and minerals [27]. In general, the results indicated that the participants ate food poor in carbohydrates, saturated fat, dietary fiber, and some vitamins and minerals. The majority of participants in the study depended on quick outside diets and did not like to eat at home, which can be considered bad food habits. 

Malnutrition manifested as overweight or underweight has been observed among Syrian children, with a high percentage of underweight in both sexes. Many factors influence the nutritional and developmental status of children, such as the socio-economic characteristics of the family, the eating habits of children, environmental factors, access to and use of health services, and hygiene practices at the household level [28]. It is well known that malnourished people spend a lot of time watching TV and playing video games, which reduces physical activity and promotes a sedentary lifestyle, affecting eating patterns and nutritional status. [17,26]. An investigation conducted in Saudi Arabia showed that the dietary problems in Saudi Arabia are mainly due to changing food habits, illiteracy, and ignorance, not a lack of food supplies or low income [29]. This finding agrees with Park et al. [30], who concluded that the primary cause of obesity and weight gain is an energy imbalance between calories consumed and calories spent, which results from changes in dietary and physical activity patterns, accompanied by environmental and societal changes related to the development and a lack of supportive policies. 

According to the present study, stunting varied between boys and girls, with a high percentage among girls. In contrast to our results, Ali et al. [26] reported that stunting is high prevailing among boys compared to girls. Prendergast and Humphrey [31] reported that stunting is a significant public health problem because of its association during childhood with poor functional outcomes such as impaired cognitive development, increased susceptibility to infection, and increased mortality risk [32]. The long-term consequences of childhood stunting include short stature, reduced work capacity, and a higher risk of poor reproductive outcomes [26]. An investigation by Fernald and Neufeld [33] suggested that stunting during childhood could increase the risk of obesity and chronic diseases. The leading causes of stunting include the mother’s poor nutritional status during pregnancy, lack of nutrition in the womb, inadequate breastfeeding, delayed complementary feeding, insufficient quality or quantity of supplementary feeding, and poor absorption of nutrients because of intestinal infections and parasites [34].

A significant and direct association of some independent variables with BMI, weight-for-age, and height-for-age indicated that the participants would gain weight and expect to be overweight or obese as well as stunted, as indicated by Ali et al. [26], who observed a direct correlation between some independent variables and BMI for Sudanese children in Saudi Arabia. Direct correlations between nutritional status and monthly household income are important factors determining the availability of food to respondents. As a result of the collected data of Syrians working in Saudi Arabia have a good income that allows them to live a decent life, and they are not expected to experience challenges connected to the interrelationships between these two factors. Previous studies [17,26] on Sudanese pupils who resided in Saudi Arabia gave similar results. Furthermore, the development in Saudi Arabia has led to the adoption of a sedentary lifestyle and the consumption of a diet high in fat and low in fiber, which is believed to be linked to poor health [17]. Accordingly, most socioeconomic characteristics and food habits as independent variables were considered risk factors and significantly and inversely correlated with the anthropometric measurements as dependent variables, with few exceptions.

## 5. Conclusions

The socioeconomic characteristics and food habits were varied between the respondents. A high percentage of participants who suffered from being underweight was observed, which was speculated to be caused by their inactive lifestyle that negatively affected their eating habits. This is indicated by a significantly low average daily intake of carbohydrates, dietary fiber, total unsaturated fat, and some vitamin and minerals compared to DRI. The hypothesized predictors of the participants’ nutritional proxies as dependent variables were significantly and inversely correlated with the participants’ socioeconomic characteristics and food habits as independent variables. The study showed that the majority of participants’ socioeconomic characteristics and food habits were risk factors.

## Figures and Tables

**Table 1:** Frequency distribution of Syrian children (boys and girls) according to socioeconomic data.

Variable	Boys		Girls	
	Frequency	Percent	Frequency	Percent
Family members <5	23	41.82	14	25.45
>5	32	58.18	41	74.55
Father’s education Illiterate	5	9.09	3	5.45
Primary	7	12.73	6	10.91
Intermediate	3	5.45	3	5.45
Secondary	18	32.73	24	43.64
Institute	0	0	2	3.64
University	22	40	17	30.91
Mother’s education Illiterate	3	5.45	2	3.64
Primary	8	14.55	5	9.09
Intermediate	4	7.27	4	7.27
Secondary	22	40	17	30.91
Institute	1	1.82	3	5.45
University	17	30.91	24	43.64
Family income <3000 SR	20	36.36	7	12.73
3000–6000 SR	22	40	30	54.54
6000–9000 SR	9	16.36	11	20.00
>9000 SR	4	7.27	7	12.73
Mothers working				
Yes	15	27.27	11	20.00
No	40	72.73	44	80.00

SR = Saudi riyals.

**Table 2:** Frequency distribution of Syrian participants (boys and girls) according to food habits.

Variable	Boys		Girls	
	Frequency	Percent	Frequency	Percent
No. of meals/day				
1	0	0	3	5.45
2	16	29.1	12	21.82
3	36	65.5	35	63.64
4	3	5.5	5	9.09
Eat breakfast				
Yes	53	96.36	54	98.18
No	2	3.64	1	1.82
Preference of food				
Yes	33	60.00	29	52.73
No	22	40.00	26	47.27
Special diet regime				
Yes	0	0	0	0
No	55	100.0	55	100.0
Vitamins and supplements				
Yes	5	9.09	7	12.73
No	50	90.91	48	87.27
Soft drinks				
No	5	9.09	3	5.45
Rarely	8	14.55	10	18.18
Sometimes	27	49.09	28	50.91
Always	15	27.27	14	25.45
Intake of sweets				
No	4	7.27	2	3.64
Rarely	6	10.91	8	14.55
Sometimes	33	60.00	28	50.91
Always	12	21.82	17	30.91

**Table 3:** The average intake of nutrients (24 h recall) concerning dietary reference intakes (DRI) for Syrian children using the *t*-test.

Items Intake	DRI	Boys			Girls		
		Mean	Difference	*p*-Value	Mean	Difference	*p*-Value
Calories (kcal)	2000.00	1110.8	−889.2	0.002 **	1005.53	−994.47	0.008 **
Protein (g)	32.00	49.27	17.27	0.007 **	46.40	14.40	0.003 **
Carbohydrates (g)	130.00	116.67	−13.33	0.010 **	127.82	−2.18	0.000 **
Dietary fiber (g)	28.00	10.17	−17.83	0.003 **	10.16	−17.84	0.005 **
Total fat (g)	65.00	38.25	−26.75	0.001 **	35.16	−29.84	0.008 **
Saturated fat (g)	20.00	14.00	−6.00	0.005 **	13.14	−6.86	0.422
Unsaturated fat (g)	45.00	170.43	125.43	0.003 **	200.00	155	0.001 **
Cholesterol (mg)	300.00	270.41	−29.59	0.009 **	250.23	−49.77	0.000 **
Vit. A µg (RE)	550.00	487.16	−62.84	0.002 **	924.12	374.12	0.005 **
Vit. B1 (mg)	0.800	0.56	−0.24	0.000 **	0.599	−0.201	0.002 **
Vit. B2 (mg)	0.800	9.21	8.41	0.003 **	0.916	0.116	0.007 **
Niacin (mg)	11.00	10.29	−0.71	0.008 **	8.46	−2.54	0.008 **
Vit. B6 (mg)	0.900	0.797	−0.103	0.006 **	0.763	−0.137	0.004 **
Vit. B12 (mg)	1.60	2.02	0.42	0.010 **	2.07	0.47	0.009 **
Vit. D (mg)	15.00	1.43	−13.57	0.002 **	1.38	−13.62	0.005 **
Vit. E (mg)	10.00	2.64	−7.36	0.000 **	2.81	−7.19	0.001 **
Folate (mg)	270.00	239.12	−30.88	0.017 *	201.73	−68.27	0.002 **
Calcium (mg)	1200.00	455.80	−744.2	0.004 **	443.05	−756.95	0.006 **
Copper (mg)	600.00	0.670	−599.33	0.001 **	8.92	−591.08	0.000 **
Iron (mg)	9.00	7.39	−1.61	0.006 **	6.73	−2.27	0.003 **
Phosphorus (mg)	1000.00	714.02	−285.98	0.000 **	964.01	−35.99	0.001 **
Selenium (mg)	37.00	62.16	25.16	0.000 **	66.86	29.86	0.007 **
Zinc (mg)	6.96	5.54	−1.42	0.003 **	5.86	−1.10	0.004 **

** *p* ≤ 0.01; * *p* ≤ 0.05. Difference = intake—DRI.

**Table 4:** Body mass index (BMI) of the Syrian participants (*n* = 110) according to WHO classification.

Interpretation		Gender		Total
		Boys	Girls	
Underweight	Frequency	12	23	35
	% within gender	21.82	41.82	31.82
Normal	Frequency	38	27	65
	% within gender	69.09	49.09	59.09
Overweight	Frequency	3	4	7
	% within gender	5.45	7.27	6.36
Obesity I	Frequency	2	1	3
	% within gender	3.64	1.82	2.73
Obesity II	Frequency	0	0	0
Obesity III	Frequency	0	0	0
Total	Frequency	55	55	110
	% within gender	100	100	100

Chi-square (*p* = 0.011). BMI classified (WHO, 1998) to underweight (BMI < 18.5), normal (BMI = 18.5–24.9), overweight (BMI = 25–29.9), or obese (BMI ≥ 30). Furthermore, obesity was subdivided to three grades: Grade 1 (BMI = 30–34.9), Grade 2 (BMI = 35–39.9), and Grade 3 or extreme obesity (BMI ≥ 40).

**Table 5:** Weight-for-age (wasting) and height-for-age (stunting) of the Syrian participants (*n* = 110) according to WHO classification system.

Interpretation		Weight-for-Height		Total	Height-for-Age		Total
		Gender			Gender		
		Boys	Girls		Boys	Girls	
Very low	Frequency	27	39	66	44	32	76
	% within gender	49.09	70.90	60.00	80.00	58.18	69.09
Low	Frequency	5	2	7	7	10	17
	% within gender	9.09	3.64	6.36	12.72	18.18	15.46
Medium	Frequency	8	5	13	2	5	7
	% within gender	14.55	9.09	11.82	3.64	9.09	6.36
High	Frequency	6	2	8	1	3	4
	% within gender	10.91	3.64	7.27	1.82	5.46	3.64
Very high	Frequency	9	7	16	1	5	6
	% within gender	16.36	12.73	14.55	1.82	9.09	5.45
Total	Frequency	55	55	110	55	55	110
	% within gender	100	100	100	100	100	100

For wasting (<2.5%) very low; (2.5 to <5%) low; (5 to <10%) medium; (10 to <15%) high; and (≥15%) very high. For stunting, (<2.5%) very low; (2.5 to <10%) low; (10 to <20%) medium; (20 to <30%) high; and (≥30%) very high. Wasting chi-square (*p* = 0.372); stunting chi-square (*p* = 0.499).

**Table 6:** Spearman correlation between socioeconomic characteristics, daily food habits, and the respondents’ anthropometric measurements of Syrian participants.

Independent Variables	Dependent Variables					
	Boys			Girls		
	BMI	Weight-for-Age	Height-for-Age	BMI	Weight-for-Age	Height-for-Age
Age	0.411 *	−0.722 **	0.222 *	0.491 **	−0.611 **	0.155 *
Family member	0.213 **	−0.025 *	−0.311 **	0.314 *	−0.344 *	−0.278 **
Father’s education	0.143 *	−0.181	−0.055 **	0.223 *	−0.114 *	−0.113 *
Mother’s education	−0.23 *	0.104	−0.143 *	−0.114	0.213	−0.212 *
Monthly income	0.124 *	−0.418 *	−0.291 **	0.195 *	−0.341 *	−0.320 **
Mother work	−0.22 *	−0.088	−0.065	−0.142	−0.223	−0.026
No. of meals/day	0.333 *	−0.052	0.530 *	0.115 *	−0.431	0.427 *
Eat breakfast	−0.23 *	−0.200 *	−0.156	−0.226 **	−0.440 *	−0.330
Prefer special food	−0.25 **	−0.075	0.163 *	-0.133	−0.235	0.291 *
Special diet regimen	−0.13 **	0.312 *	−0.061	−0.115 *	0.323 *	−0.196 **
Intake of supplements or vitamins	0.099	−0.139	0.290 *	0.038	−0.225	0.311 *
Intake of soft drinks	0.175 *	−0.500 **	0.145	0.284 *	−0.167 *	0.224 *
Eat sweets	0.614 **	−0.371 *	0.274 *	0.664 **	−0.294 *	0.311 *

* *p* ≤ 0·05, ** *p* ≤ 0·01.

## Data Availability

The datasets used and analyzed in the current study are available from the corresponding author on reasonable request.
